# The ClaDS rate-heterogeneous birth–death prior for full phylogenetic inference in BEAST2

**DOI:** 10.1093/sysbio/syad027

**Published:** 2023-05-10

**Authors:** Joëlle Barido-Sottani, Hélène Morlon

**Affiliations:** Institut de Biologie de l’ENS (IBENS), École normale supérieure, CNRS, INSERM, Université PSL, 75005 Paris, France; Institut de Biologie de l’ENS (IBENS), École normale supérieure, CNRS, INSERM, Université PSL, 75005 Paris, France

**Keywords:** BEAST2 package, birth–death, ClaDS model, phylogenetic inference, rate heterogeneity, tree prior

## Abstract

Bayesian phylogenetic inference requires a tree prior, which models the underlying diversification process that gives rise to the phylogeny. Existing birth–death diversification models include a wide range of features, for instance, lineage-specific variations in speciation and extinction (SSE) rates. While across-lineage variation in SSE rates is widespread in empirical datasets, few heterogeneous rate models have been implemented as tree priors for Bayesian phylogenetic inference. As a consequence, rate heterogeneity is typically ignored when reconstructing phylogenies, and rate heterogeneity is usually investigated on fixed trees. In this paper, we present a new BEAST2 package implementing the cladogenetic diversification rate shift (ClaDS) model as a tree prior. ClaDS is a birth–death diversification model designed to capture small progressive variations in birth and death rates along a phylogeny. Unlike previous implementations of ClaDS, which were designed to be used with fixed, user-chosen phylogenies, our package is implemented in the BEAST2 framework and thus allows full phylogenetic inference, where the phylogeny and model parameters are co-estimated from a molecular alignment. Our package provides all necessary components of the inference, including a new tree object and operators to propose moves to the Monte-Carlo Markov chain. It also includes a graphical interface through BEAUti. We validate our implementation of the package by comparing the produced distributions to simulated data and show an empirical example of the full inference, using a dataset of cetaceans.

Time-calibrated trees represent the evolutionary relationships between species or organisms as well as a timescale of these relationships therefore providing a crucial basis for hypothesis-testing in the life and earth sciences. Phylogenetic inference uses molecular alignment data to estimate phylogenetic trees and can be implemented in a Maximum Likelihood or Bayesian framework. One specificity of Bayesian phylogenetic inference is that it includes a model of the evolutionary process that gave rise to the phylogeny, also called a tree prior. In full phylogenetic inference, the tree prior and the phylogeny are estimated jointly from molecular data (Bouckaert et al. 2019).

Tree priors are generally divided into two main classes, namely coalescent processes and birth–death processes. This second category assumes that the tree process is driven by two main evolutionary rates, namely the birth rate, which describes the rate at which new lineages arise in the phylogeny, and the death rate, which describes the rate at which lineages are removed from the phylogeny (MacPherson et al. 2021). Birth—death models are also widely applied directly to a previously estimated phylogeny to infer the underlying evolutionary process (Pennell and Harmon 2013; Stadler 2013; Morlon 2014).

The simplest birth–death models are homogeneous, meaning that they have identical birth and death rates across lineages. However, lineage-specific variation in birth and death rates is generally assumed to be widespread in empirical datasets, and many morphological and ecological traits have been proposed to contribute to this variability, such as body size (Inostroza-Michael et al. 2018) or environmental conditions (Letsch et al. 2018). Thus, many birth–death processes have been developed to model across-lineage variation in rates, including the State-dependent Speciation and Extinction (SSE) family of models (Maddison et al. 2007; FitzJohn 2012), which have primarily been used with fixed phylogenies or the multi-type birth–death processes (Kühnert et al. 2016; Barido-Sottani et al. 2020; Höhna et al. 2019), which can also be used as priors in full phylogeneticinference.

Unlike these previous models, which focused on identifying large shifts in evolutionary rates, the cladogenetic diversification rate shift (ClaDS) model is designed to model small progressive changes in evolutionary rates throughout a phylogeny (Maliet et al. 2019). Implementations of ClaDS have demonstrated that the model can reliably detect rate heterogeneity on simulated phylogenies, as well as recover accurate rate estimates (Maliet et al. 2019; Maliet and Morlon 2021). ClaDS has also been shown to provide higher marginal likelihoods than other heterogeneous rate models on empirical datasets (Ronquist et al. 2021). The specificity of the ClaDS model compared to other birth–death processes is that each edge in the phylogeny is associated with its own birth and death rates. At each birth event, new birth and death rates are sampled from the birth and death rates of the parent edge, following a lognormal distribution. This is similar in principle to a relaxed autocorrelated clock model (Thorne et al. 1998).

Previous implementations of ClaDS have focused on inferring the parameters of the model from a fixed phylogeny (Maliet et al. 2019; Maliet and Morlon 2021; Ronquist et al. 2021). However, using a fixed phylogeny does not account for the uncertainty associated with the phylogeny reconstruction, which is problematic for datasets with large uncertainties. In addition, phylogenies present in the literature are frequently inferred under the assumption that evolutionary rates are constant and homogeneous, which leads to biases in their dating (Ritchie et al. 2022) and possibly in their topology, and subsequent biases in the inference of the evolutionary process (Condamine et al. 2015). Full phylogenetic inference allows the phylogeny inference to properly account for potential rate variations and naturally integrates phylogenetic uncertainty in the posterior distributions. We present here our new ClaDS package for the popular Bayesian phylogenetic inference framework BEAST2 (Bouckaert et al. 2019), designed to perform full phylogenetic inference using the ClaDS model as a tree prior.

## The ClaDS Package

### Parametrization

In the ClaDS model, the birth–death process starts with an initial birth rate at the root $\lambda_{0}$. At each birth event, new birth and death rates are sampled for the descendant edges from the birth and death rates of the parent edge. The new birth rates are sampled from a lognormal distribution, using a trend parameter $\alpha_{\lambda}$ and a variance parameter $\sigma_{\lambda}$. Thus, if lineages $N_{1}$ and $N_{2}$ descend from lineage $N$ (with birth rate $\lambda_{N}$), the corresponding birth rates are sampled as $\lambda_{1},\lambda_{2}\sim LogNormal(\alpha_{\lambda }\lambda_{N},\sigma_{\lambda })$. Our ClaDS package allows death rates to be sampled in two different ways: 1) using a turnover parameter $\epsilon={\mu}/{\lambda}$ which is constant throughout the tree, or 2) following a lognormal sampling process analogous to the process used for birth rates, with parameters $\mu_{0}$, $\alpha_{\mu}$, and $\sigma_{\mu}$. The first parametrization corresponds to the ClaDS2 model presented in Maliet et al. (2019), while the second has not been previously implemented. The death rate parametrization is chosen by the user and cannot be changed throughout the inference. Either birth or death rates (or both) can also be set to be constant throughout the entire phylogeny, by fixing the corresponding $\alpha=1$ and $\sigma=0$. When applied to the death rate, this corresponds to the ClaDS1 model from Maliet et al. (2019), or the ClaDS0 model if $\mu_{0}=0$. The best choice of parametrization will depend on the dataset, and on what characteristics are likely to be driving the rate variations: the turnover parametrization is more appropriate when both birth and death rates are jointly affected by similar variations, whereas the lognormal parametrization corresponds to a scenario where birth and death rate variation are decoupled or where only one rate is assumed to vary. Alternatively, model selection could be used to decide between both parametrizations, although we have not tested this method with our current package.

Finally, our ClaDS package contains a sampling parameter $\rho$, which represents the probability for each extant species to be sampled at present, taken to be identical across species. All these parameters can be estimated by the inference; however, at least one of $\rho$, $\lambda_{0}$, and $\mu_{0}$ or $\epsilon$ has to be fixed for the model to be identifiable (Stadler 2009). In most macroevolution studies, we expect that $\rho$ will be the most well-known parameter and thus the easiest to fix to the correct value. Note that fixing $\lambda_{0}$ or $\mu_{0}$ will only constrain the rates at the root of the tree. In particular, it will not result in constant rates throughout the tree. Similarly, fixing $\epsilon$ will constrain the ratio of birth and death rates, but not the values of the rates themselves.

### Data Augmentation

Similar to Maliet and Morlon (2021), our implementation relies on data augmentation. Instead of directly calculating the likelihood of the reconstructed tree under the model, we sample the complete tree and calculate its likelihood. Both the reconstructed tree and the complete tree are estimated in the MCMC; however, complete trees are sampled by conditioning on the current value of the reconstructed tree. In our implementation, the complete tree is split into subtrees. Each subtree is associated with an edge of the reconstructed tree and contains this edge as well as all the unsampled lineages that derive from this edge (see Figure 1 in Maliet and Morlon (2021) for a representation of subtrees). The augmented tree is represented by the AugmentedTree class, which stores the complete subtree associated with each edge of the reconstructed tree, as well as the simulated birth and death rates for all nodes of the complete tree. Complete subtrees use the AugmentedNode class, an extension of the regular Node class that also stores the birth and death rates for each node.

**Figure 1:**
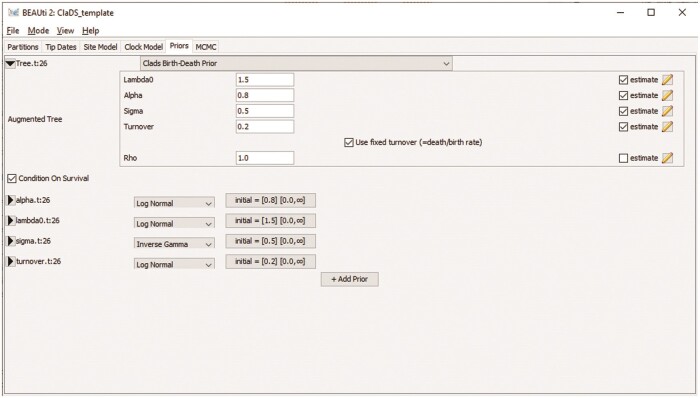
Screenshot of the ClaDS tree prior setup in the BEAUti graphical user interface, with the turnover parametrization selected. All parameters except the extant sampling proportion $\rho$ are set to be estimated.

Complete subtrees are simulated using a Gillespie forward sampling algorithm (Gillespie 1976). As the complete tree is always simulated, given a specific reconstructed tree, we assume that the reconstructed tree is fixed throughout the simulation process. The simulation starts with one lineage, whose birth and death rates are sampled from the ancestral birth and death rates, or from the initial birth and death rates if simulating from the root of the tree. The simulation then proceeds according to the ClaDS model. At each step of the simulation, a new event time is drawn from an exponential distribution with a rate equal to the sum of birth rates and death rates for all lineages currently extant in the subtree, and the event is chosen according to its respective probability. If the event is a birth, a parental lineage is chosen at random and two descendant lineages are created with birth rates and death rates sampled from the ancestral rates. If the event is a death, a lineage chosen at random is removed. Once the process reaches the end time of the edge, one of the surviving lineages is selected uniformly at random to serve as the lineage appearing in the reconstructed phylogeny. All other lineages are considered unsampled. If the end time is not at the present, the unsampled lineages are simulated further until the process goes extinct or reaches the present. A simulated subtree that goes extinct before the end time of the edge, or which is incompatible with the reconstructed tree (for instance, $\rho=1$, but some unsampled lineages survive until the present) is considered invalid. To ensure that the inference can always be started, a default subtree is provided if the complete subtree simulation fails during the initialization phase of the inference. This default subtree simply assumes that the complete tree matches the reconstructed tree exactly.

The package can output the full posterior distribution of complete trees in addition to the posterior distribution of reconstructed trees, allowing the sampled complete trees to be used in further analyses. Due to the size of the resulting file, this option is not active by default, however the package includes an example XML showing how to output the augmentedtrees.

### Operators

Bayesian inference in BEAST2 relies on a Monte-Carlo Markov chain (MCMC) algorithm, which uses a random walk through the parameter space to explore the posterior distribution. An important component of the algorithm is the operators, which propose new positions in the chain. Any parameter or object estimated by the inference is associated with at least one operator; at each step of the chain, one operator is selected at random among all the operators configured for the analysis, and proposes a change to its corresponding parameters. To ensure the convergence of the MCMC, these operators are balanced according to the Metropolis–Hastings algorithm, which adds an operator-specific factor (the Hastings ratio) to the acceptance probability of theproposal.

The previous implementations of ClaDS only included operators to perform changes on the numerical parameters of the model (Maliet and Morlon 2021). Since our new implementation includes the full phylogenetic inference, it also requires operators for the tree. The augmented tree is conditioned by the reconstructed tree, thus any operator proposing changes to the reconstructed tree needs to also perform the appropriate changes to the augmented tree. In consequence, we extend the default tree operators used by BEAST2 (first introduced in Drummond et al. (2002)) to augmented versions (Augmented Wilson Balding, Augmented Subtree Exchange, and Augmented Node Shift), which are included in the ClaDS package. When the height or position of a node $N$ in the reconstructed tree is changed by any of these operators, at most three of the augmented subtrees need to be resimulated: the subtree associated with the edge above node $N$, and the two subtrees associated with the edges directly descending from node $N$ if $N$ is not a tip. The resampled subtrees are simulated following the Gillespie algorithm outlined in the previous section. If any of the simulations involved in an operator leads to an invalid subtree, the entire proposal is automatically rejected and the inference moves on to the next step. Otherwise, the logarithm of the Hastings ratio (logHR) associated with the resimulation of the complete subtrees is calculated as the log probability of the previous subtree minus the log probability of the new subtree (see Maliet and Morlon (2021) for the full calculation of the Hastings ratio). This value is added to the logHR of the tree proposal. The complete subtrees associated with branches of the reconstructed tree that have not been modified are notresampled.

We also provide another operator focused onsampling the complete tree, the AugmentedTreeSimulationOperator, which chooses one edge of the reconstructed tree uniformly at random and resimulates the augmented subtree associated with that edge. The process and ratio calculation are similar to the other operators, except that the reconstructed tree is notmodified.

BEAST2 already includes standard operators for numerical parameters such as the parameters of the rate distributions (e.g. $\lambda_{0}$, $\alpha_{l}$), and our package did not modify these operators. As a result, the augmented subtrees are not resampled when proposing a new value of these parameters.

### Graphical Interface

As shown in Figure 1, the ClaDS package is fully integrated into the BEAUti graphical user interface, which allows users to easily configure analyses using ClaDS as a tree prior. The interface provides a default configuration for the model and the prior distributions of the estimated parameters, as well as a default operator setup using the new augmented tree operators. The parametrization of the death rate, the set of parameters to estimate, and the initial values and priors on all estimated parameters can be adjusted by the user.

The ClaDS package is fully compatible with all other functions of the BEAUti interface, such as using multiple alignment partitions, customizing the substitution and clock models, and selecting a starting tree. It can also be used in combination with node age constraints derived from fossil data, although directly integrating fossil samples is currently not implemented.

## Validation

**Figure 2:**
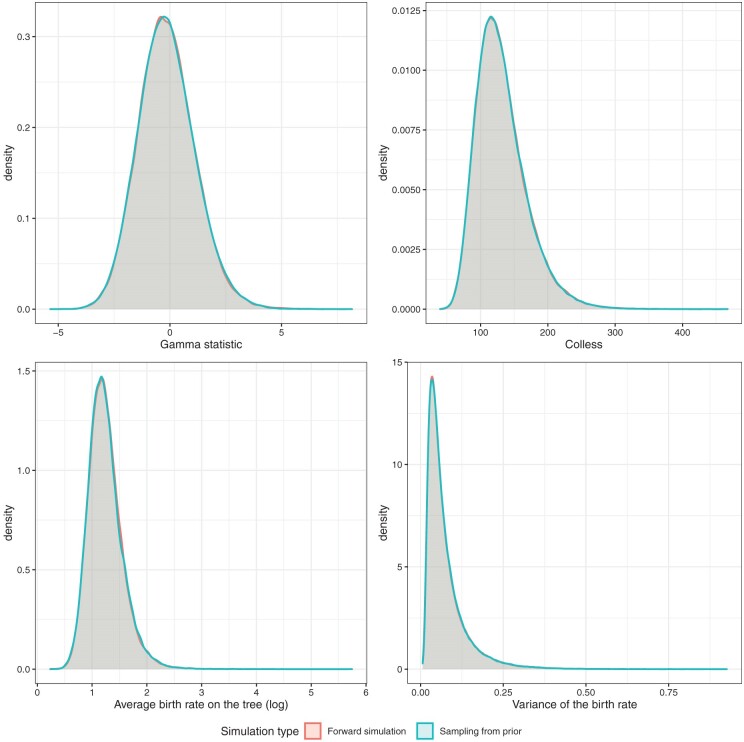
Posterior distributions of the Gamma statistic, the Colless index, the average birth rate across the reconstructed tree, and the birth rate variance parameter. The distribution shown in green was obtained by sampling from the prior, whereas the distribution in red resulted from simulating forward under the ClaDS model. The average birth rate is plotted on a log scale. For colour figure refer to the online version.

We validated our implementation of the ClaDS model by comparing the tree and parameter distributions obtained from 1) sampling from prior, that is, running the MCMC inference without data, and 2) simulating under the model by using a forward sampling algorithm. If the implementation in the package matches the simulation model, then both distributions should be identical. This “sampling from the prior” procedure has been described in Vaughan et al. (2014), and allows us to confirm that both the likelihood function and the operators work as expected. The distributions were calculated based on a sample of 100,000 trees for both the simulation and the inference. The priors used to sample the model parameters are listed in the Supplementary materials. We evaluated four different measures, namely the Gamma statistic (Pybus and Harvey 2000), the Colless index (Colless 1982), the average birth rate across the reconstructed tree, and the birth rate variance parameter $\sigma_{\lambda}$. Figure 2 shows the comparison of both distributions. Since the distributions match perfectly, we are confident that our implementation of the ClaDS model is correct.

## Empirical Example

As an empirical example, we used the alignment provided by Steeman et al. (2009), which contains sequences for 6 mitochondrial and 9 nuclear genes for 87 of 89 extant cetacean species. We excluded from our analysis the three outgroup taxa that were present in the original alignment, as outgroup taxa are not needed in Bayesian phylogenetic inference. The full alignment thus contains 87 sequences with 16 175 characters each. The substitution and clock models were set similarly to Barido-Sottani et al. (2019). To simplify the analysis and reduce the uncertainty in the estimated divergence times, we did not use node age calibrations but instead fixed the root age of the tree to 44 Ma, in accordance with results from Barido-Sottani et al. (2019).

The tree prior was set to the ClaDS model, using our new package. The death rate parametrization was set to use the turnover as parameter, meaning that the turnover was constant throughout the tree. The extant sampling proportion was set to $\rho=0.98$, and all other parameters were estimated. Prior distributions and operators were set to the default values. The MCMC chain was run for 600,000,000 iterations, and convergence was assessed using Tracer v1.7 (Rambaut et al. 2018). All measures reached effective samples sizes (ESS) values $>200$, with the exception of one alignment partition (out of 28) which reached an ESS $>100$. The analysis took about 12 days of computation time on a 1-core CPU, without using the BEAGLE library.

**Figure 3:**
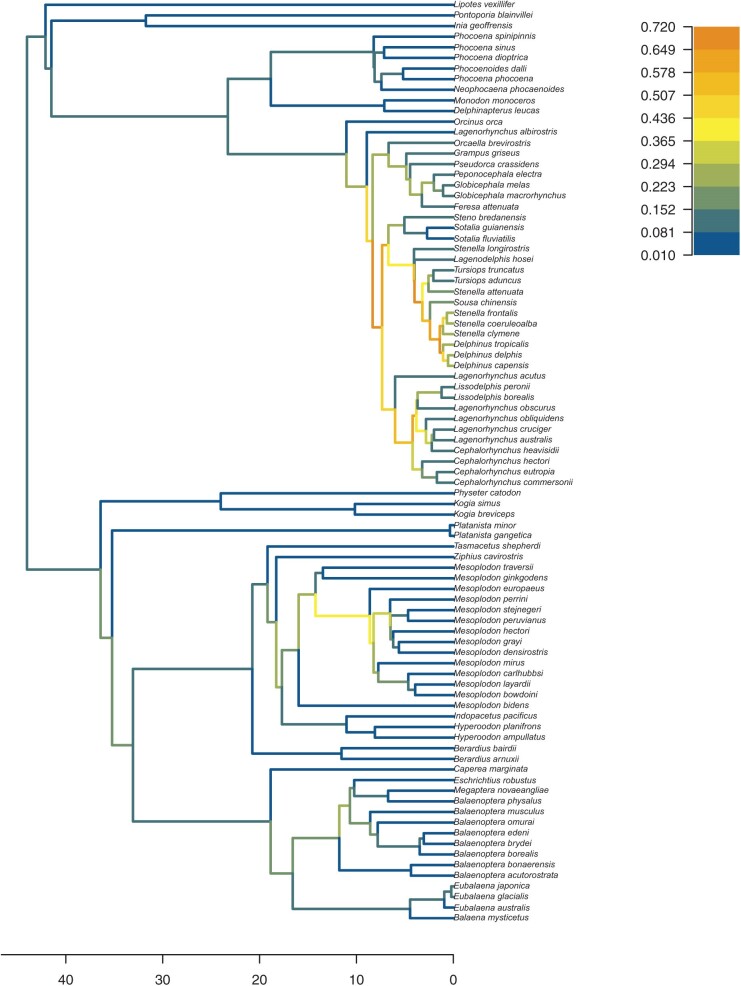
Estimated MCC tree for the Cetaceans clade from a Bayesian full phylogenetic inference using the ClaDS tree prior. Edges are colored by their estimated median birth rate $\lambda$.

Figure 3 shows the Maximum Clade Credibility (MCC) tree obtained using TreeAnnotator with a burn-in percentage of 30%, colored by the estimated median birth rate for each edge. The median estimates for the ClaDS parameters are $\lambda_{0}=0.14$, $\alpha_{\lambda}=0.69$, $\sigma_{\lambda}=0.90$, and $\epsilon=0.61$. The mean rate variation at birth, $m_{\lambda}=\alpha_{\lambda}\times exp(\sigma_{\lambda}^{2}/2)$ is thus estimated as $m_{\lambda}=1.03$, which corresponds to a slight increasing trend in birth rates from the root toward the tips of the tree.

Most of the tree is assigned as a median birth rate between $\approx 0.01$ and $\approx 0.15$. Edges that constitute the backbone of the *Delphinidae* family are inferred to have higher birth rates, which match the higher diversity of this subclade compared to the rest of the tree.

Supplementary Figures S1 and S2 show the same analysis under, respectively, a constant birth–death tree prior, and the multi-type birth–death prior implemented in the BEAST2 package Multi-States Birth-Death (MSBD). All three analyses recover similar estimates for the average birth rate across the tree. The inference using MSBD also finds higher birth rates in the backbone of the *Delphinidae* family, as well as slightly higher birth rates on edges close to the root of the tree, coherent with the findings of the inference using ClaDS. However, estimated birth rates for the *Delphinidae* family are lower under the MSBD tree prior than under the ClaDS tree prior. This is likely due to the different assumptions between the models: as MSBD assumes that rates are inherited at birth events, it is less likely than ClaDS to infer sharp differences in rates between adjacent edges.

The MCC tree topology obtained by the three different analyses also shows some differences, particularly in the *Mesoplodon* genus, both in topology and in the estimated node ages. This confirms that the use of a different tree prior can impact the reconstructed phylogeny in Bayesian inferences.

## Discussion

In contrast with other existing multi-type birth–death processes, the ClaDS model is able to represent the gradual evolution of evolutionary rates throughout a phylogeny, using a small number of parameters to represent the general process. Previous implementations of ClaDS have, however, been limited by their reliance on a fixed phylogeny, as well as limited options for configuring estimated parameters and prior distributions.

Integrating ClaDS with the BEAST2 framework allows it to be combined with existing substitution and clock models in order to infer phylogenies from a molecular alignment while properly accounting for rate heterogeneity in the evolutionary process. A recent simulation study has shown that not accounting for such heterogeneity can result in biases in molecular dates (Ritchie et al. 2022). Mismatches between the tree prior and the actual evolutionary process could also potentially bias the reconstructed topologies toward shapes that are more consistent with the assumed homogeneous and constant rates, although this has not been demonstrated in the literature. Our comparison of three different tree priors on the Cetaceans clade shows that the choice of model can indeed affect the recovered phylogeny. The ClaDS package will allow us to explore this issue further by comparing the rate estimates obtained from phylogenies estimated under either a homogeneous- or heterogeneous-rate tree prior. Finally, the integration of ClaDS within BEAST2 makes the model more accessible to users who are already familiar with BEAST2 and its interface. Finally, it greatly increases its flexibility by making use of the configuration options available in BEAST2, such as the different prior distributions or input and outputformats.

The performance of full phylogenetic inference on our empirical example, in terms of computation time, appears comparable to other complex birth–death processes in BEAST2, such as the multi-type birth–death process implemented in the BDMM package (Kühnert et al. 2016). One potential area of improvement would be to increase the acceptance rate of augmented tree operators. In the current implementation, the simulation of an invalid augmented subtree in any part of an operator leads to the rejection of the entire proposal, which leads to low acceptance probabilities for our new augmented operators ($\approx 0.03$ in the Cetaceans example, as opposed to $\approx 0.25$ for the operators on the numerical parameters). The number of steps required to achieve convergence of the model is thus higher than for models without data augmentation. Another important caveat of the current implementation is that the size of the augmented tree compared to the reconstructed tree will increase as the sampling probability $\rho$ decreases, which will strongly impact the performance of the model on datasets with low sampling fractions. Future work on the package will include the implementation of the ClaDS likelihood calculation using numerical integration (Maliet et al. 2019), which will allow the package to be used on a wider range ofapplications.

In the future, implementing ClaDS into the BEAST2 ecosystem will also allow the model to be combined with other models of the framework, such as the Fossilized Birth–Death process for trees with fossil specimens (Heath et al. 2014), included in the SA package (Gavryushkina et al. 2014).

## Data Availability

The ClaDS package is available for BEAST 2.6 and BEAST 2.7 through the package manager (see installation instructions here: https://www.beast2.org/managing-packages/) and as a public Git repository (https://bitbucket.org/bjoelle/clads/). The R code used for simulation and validation, the BEAST2 XML files used to run the validation and empirical example, and the results of the validation are available on Data Dryad, https://doi.org/10.5061/dryad.0vt4b8h2g.

## Supplementary Material

Data available from the Dryad Digital Repository: https://doi.org/10.5061/dryad.0vt4b8h2g.
